# Analysis of Conization Results in Patients undergoing Hysterectomy for Uterine Adenocarcinoma

**DOI:** 10.1055/s-0040-1709191

**Published:** 2020-05

**Authors:** Denise Gasparetti Drumond, Isabel Cristina Gonçalves Leite, Vivian de Oliveira Rodrigues, Gabriel Duque Pannain, Miralva Aurora Galvão Carvalho, Renata Guimarães Rabelo do Amaral

**Affiliations:** 1 Department of Surgery, Universidade Federal de Juiz de Fora, Juiz de Fora, MG, Brazil

**Keywords:** conization, hysterectomy, adenocarcinoma in situ, conização, histerectomia, adenocarcinoma in situ

## Abstract

**Objective** To observe if the histopathological result of a conization performed after cervical adenocarcinoma in situ diagnosis is compatible with the histopathological analysis of a subsequent hysterectomy.

**Methods** The present descriptive and observational research consisted of the analysis of the medical records of 42 patients who were diagnosed with in situ adenocarcinoma postconization. The analysis consisted of whether there was compatibility between the histopathological reports of conization and hysterectomy and if there was an association between adenocarcinoma in situ and another neoplasia (squamous disease). Interpretation of any immunohistochemistry reports obtained was also performed. In addition, clinical and epidemiological data were also analyzed.

**Results** A total of 42 conizations were performed, 33 (79%) were cold knife conizations and 9 (21%) were loop electrosurgical excision procedures (LEEPs). Of the patients analyzed, 5 (10%) chose not to undergo subsequent hysterectomy to preserve fertility or were < 25 years old. Out of the 37 patients with adenocarcinoma in situ who underwent subsequent hysterectomy, 6 (16%) presented with residual disease. This finding proved incompatible with the finding of the conizations, which had ruled out invasive cancer.

**Conclusion** The prevalence of adenocarcinoma in situ increased in the past years. There is still a large part of the medical literature that advocates the use of conservative treatment for this disease, even though it is common knowledge that it is a multifocal disease. However, the majority of studies advocate that hysterectomy should remain the preferred treatment for women who have already completed their reproductive purpose.

## Resumo

**Objetivo** Observar se o resultado proveniente de uma conização realizada após o diagnóstico de adenocarcinoma cervical in situ é compatível com a análise histopatológica da histerectomia.

**Métodos** A pesquisa foi descritiva e observacional e consistiu na análise de prontuário de 42 pacientes que tiveram o diagnóstico de adenocarcinoma in situ obtidas por conização. Foram analisados se havia compatibilidade entre os laudos de conização e histerectomia, margens do cone, se havia associação com outra patologia (doença escamosa) e interpretação de eventuais laudos histoquímicos obtidos. Além disso, também foram analisados dados clínico-epidemiológicos.

**Resultados** Foram realizadas 42 conizações, sendo 33 (79%) por cone clássico e 9 (21%) por cirurgia de alta frequência. Das pacientes analisadas, 5 (10%) não foram submetidas a histerectomia por desejarem manter a fertilidade ou por terem idade < 25 anos. Das 37 pacientes com adenocarcinoma in situ no exame prévio realizado e que foram submetidas à histerectomia posteriormente, 6 (16%) apresentaram doença residual após o procedimento cirúrgico, apresentando laudos do anatomopatológico pós-histerectomia incompatíveis com o achado na conização que atestava margens livres.

**Conclusão** A prevalência do adenocarcinoma in situ vem aumentando cada vez mais. Ainda há uma grande parte da literatura que defende o uso do tratamento conservador para esta doença, mesmo sabendo que ela é uma doença multifocal e que pode estar presente mesmo em situações nas quais o anatomopatológico evidencie margens livres. Tendo em vista essas características, a maioria preconiza que a histerectomia continua a ser o tratamento preferencial nas mulheres que já completaram o seu intuito reprodutivo.

## Introduction

Invasive cervical cancer is the 3^rd^ most common cancer and the 4^th^ cause of cancer-related death in women worldwide.^1^ More than 500,000 new cases are estimated per year, resulting in ∼ 265,000 deaths each year.^2^ In 2016 in the United States, 12,990 new cases of cervical cancer were diagnosed, while in Brazil there were ∼ 15,590 new cases in 2014, representing the 2^nd^ most common cancer in females.^3^ ^4^

In 80% to 90% of cases, the identified subtype is squamous cell carcinoma, while in 10 to 20% it is adenocarcinoma.^5^ While the incidence of squamous cell carcinoma has decreased worldwide, adenocarcinomas have become increasingly common.^5^ ^6^ The increased prevalence of this neoplasm is related to a higher use of diagnostic methods, such as cytopathology, colposcopy and biopsy.^7^

When compared with squamous cell carcinoma, adenocarcinoma has a worse prognosis and mortality rates that have remained relatively stable over the past 3 decades. These constant rates point to failures in screening and early detection of cervical cancer precursor lesions, which results in diagnosis at an advanced stage of the disease and, consequently, worse survival.^8^ Cervicovaginal cytology, despite having a high sensitivity and specificity for detecting squamous carcinoma, has a low sensitivity for detecting adenocarcinoma.^9^

These difficulties are due to the fact that endocervical cells are highly cohesive, which limits smear collection. In addition, there is a difficulty in making cytopathological differentiation between glandular atypia and benign changes such as metaplasia, Arias-Stella reaction, polyps, or cervical endometriosis.^10^ An identification of atypical glandular cells (AGC) is not a very common finding (accounting for 0.2% of cytologies), and may only mean the presence of benign uterine pathology.^11^ In colposcopy, the morphology of precursor lesions is also poorly defined, often presenting subtle alterations. Some lesions may be hidden in the endocervical canal and, in 15% of cases, a multifocal pattern, with noncontiguous lesions interspersed with normal epithelium, can be identified.^12^

The precursor lesion of adenocarcinoma is in situ adenocarcinoma, with an important causal relationship with human papillomavirus (HPV) infection and use of hormonal contraceptives.^13^ However, unlike squamous cell carcinoma, differentiating in situ adenocarcinoma from invasive adenocarcinoma on cytology is complex and often unfeasible.^14^ Nevertheless, there is still no other cost-effective method for detecting cervical adenocarcinoma other than cytology.^9^

After cytological alteration, the patient in question should be submitted to a histopathological analysis, which may be through a surgical procedure or high frequency surgery, with no superiority in one method over the other.^15^ ^16^ When histopathological analysis results in adenocarcinoma in situ, physicians should choose between considering the patient as treated or if the patient should undergo a hysterectomy.^17^ Such a choice is difficult and should be individualized for each patient, as the literature is controversial in this regard, arguing that treatment should be hysterectomy, given the risk of recurrence and the risk of invasive cancer, whereas conization should be considered the treatment of choice in nulliparous patients, allowing future fertility.^15^ ^17^

Approximately 48 to 69% of women with reports suggestive of adenocarcinoma in situ have confirmed lesion on histopathology examination; of this percentage, 38% still have invasion report.^18^ In addition, the literature shows that patients with free margins in conization have a chance of having invasive disease and future recurrence.^19^ The present research aims to demonstrate the correlation between the finding of adenocarcinoma in situ in conization and what was found in the anatomopathological examination of fragment after hysterectomy.

## Methods

The present research was descriptive and observational and consisted of the analysis of the medical records of 42 patients who had diagnosis of in situ adenocarcinoma obtained by conization, either obtained by classic cone or by high frequency surgery. Data were obtained from pathological anatomy laboratories in the city of Juiz de Fora, state of Minas Gerais, Brazil, from 2010 to 2019. Through the result of cervical adenocarcinoma in situ obtained in the anatomopathological examination, we searched for histopathological reports of all of those who underwent hysterectomy. We analyzed whether there was compatibility between the reports of conization and hysterectomy, margins of the cone, if there was association with another pathology (squamous disease) and interpretation of any histochemical reports obtained. In addition, we analyzed clinical-epidemiological data, such as age, menarche, sexarche, number of sexual partners, use of hormonal contraception, number of pregnancies and deliveries, whether or not a smoker, result of serology for HIV, syphilis, Hepatitis (B and C) and symptoms at time of diagnosis. The project was approved by the local ethics committee under the opinion number 3,079,564.

## Results

**Fig. 1:**
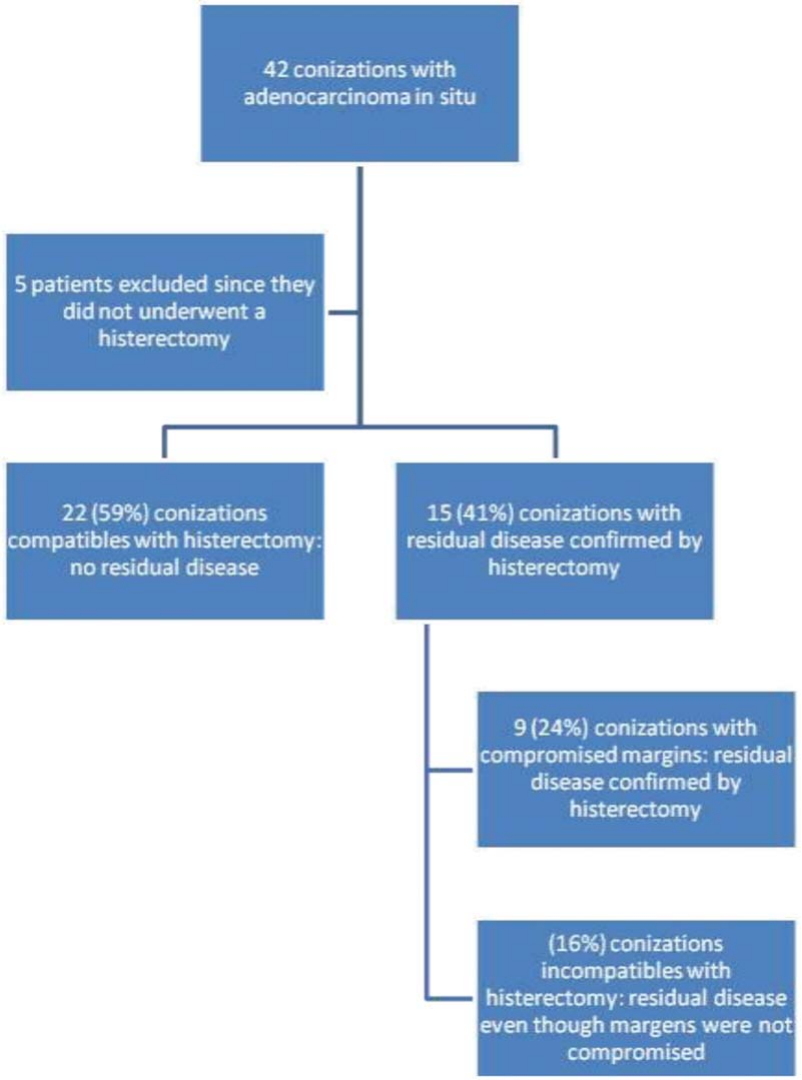
Diagnostic method for adenocarcinoma.

In the present research, regarding the diagnostic method for adenocarcinoma, 42 connections were performed, 33 (79%) by classic cone and 9 (21%) by high frequency surgery. Out of the 42 patients analyzed, 5 (10%) were not submitted to hysterectomy, and of these 5, 4 were not submitted to surgical procedure because they wanted to maintain fertility, and 1 patient was only 25 years old, and a more conservative approach was chosen (Fig. 1).

Out of the 37 patients with adenocarcinoma in situ in a previous exam who underwent hysterectomy afterwards, 15 (41%) presented residual disease confirmed by hysterectomy. Of these, 9 (24%) patients had conizations with compromised margins, and the other 6 (16%) conizations were incompatible with the previous report, presenting residual disease even though the conization was diagnosed with adenocarcinoma in situ with uncompromised cervical margins.

Out of the 9 (21%) patients who had compromised cervical margins, 4 (44%) had endocervical margin, 3 (33%) ectocervical margin and 2 (22%) had both compromised margins. In all of these patients, hysterectomy confirmed residual disease. In addition, of the patients who underwent conization, 4 (8%) were associated with cervical intraepithelial neoplasia. Nine (18%) reports suggested immunohistochemistry stating that it was not possible to safely establish whether the specimen had in situ or invasive adenocarcinoma, and could not unambiguously establish whether the origin was endometrial or endocervical.

For clinical and epidemiological analysis of the patients, only 31 medical records were analyzed because the others were excluded due to absence of data.

The average age of patients analyzed was 45.55 years old (95% confidence interval [CI]: 45.13–45.97), with the youngest age being two 25-year-old patients and the oldest being a 77-year-old patient. The average age of menarche of patients analyzed was 12.2 years (95%CI: 11.8–12.5), with the youngest age 9 years and the oldest 15 years. The mean age of sexarche was 20.5 years (95%CI: 20.3–20.7), the youngest being 15 and the oldest at 30 years old.

The analysis of the number of partners, as well as the use of contraception were impaired due to the heterogeneity of the medical records studied, and the average number of partners found were 3, being the lowest 1 and the largest 7. A total of 5 (16%) patients were using contraception. A total of 3 (10%) patients reported using condom for contraception. On others reports, use of contraception was denied.

The average number of pregnancies presented by the patients was 2.85 (95%CI: 2.82–2.88) pregnancies, with the lowest number 0 and the highest 9, similarly to the average number of deliveries, that was 2.5 (95% CI: 2.4–2.6) deliveries, the lowest being 0 and the highest 9.

All of the patients analyzed had negative serology for HIV, syphilis, hepatitis B, C and E. Only 4 (13%) patients were smokers.

From the medical records analyzed, it was found that only 7 (23%) patients had symptoms, and of these patients, 5 (71%) had abnormal uterine bleeding and 2 (29%) had sinusorragia.

## Discussion

Cervical adenocarcinoma is a histological diagnosis made from local biopsy, and can be made by several techniques, such as directed by colposcopy, endocervical curettage or conization.^20^ The conization can be performed by several techniques, and although there is no evidence in literature that the technique used interferes with the outcome of the condition, classical conization is preferable to high-frequency surgery because it provides a more complete material, easier for pathological analysis. The European Society of Gynaecological Oncology (ESGO) recommends that in women desiring fertility preservation, loop or laser conization are preferable to cold-knife conization.^21^ ^22^ ^23^

Given its incidence in young women, conservative treatment has been increasingly viewed as a therapeutic option. However, doubt and controversy persist as to the feasibility and safety of conservative treatment in women with this condition. Adenocarcinoma in situ has been described over the years as a multifocal disease, with high distribution in the endocervical canal and with a high risk of occult carcinomas and where negative margins play a limited role in predicting residual lesions.^24^

Therefore, total hysterectomy has been the gold standard treatment. Authorities in this theme such as the European Society for Medical Oncology (ESMO), ESGO, the American College of Obstetricians and Gynecologists (ACOG) and the National Comprehensive Cancer Network (NCCN) recommend that only women who wish to maintain fertility should not undergo this treatment. The ESMO and ESGO also recommend explicitly that procedures such as trachelectomy must be considered in patients who wish to maintain fertility.^25^ ^26^ ^27^ This group of women represents 10% of the patients analyzed in our study and they all underwent conization and are closely monitored by the attending physician, as the literature recommends.^28^ ^29^ ^30^

However, what is also discussed regarding conservative treatment of adenocarcinoma is the concern with the follow-up of these patients. Cervicovaginal cytology does not have the same accuracy in detecting glandular lesions as it does for high-grade squamous lesions. But it still remains as the preferred complementary exam in diagnosis and surveillance of disease recurrence after conization.^17^

Recent studies appear to show the importance of HPV testing in predicting disease recurrence. A study of 166 conservatively-treated adenocarcinoma in situ patients showed that presence of high-risk HPV during follow-up is the most important independent predictive factor for recurrence and progression to invasive adenocarcinoma.^31^

A 2009 meta-analysis assessed the risk of residual or recurrent glandular preinvasive disease after conization. Repeating the procedure in 607 patients, positive margins were associated with a 19.4% increase in risk for residual disease, and in this same study, it was noticed that even patients with free surgical margins had a chance of 2.6% to have residual disease.^32^ The ESGO suggests that in case of positive margins, a repeat conization should be performed to rule out more extensive invasive disease, since that even with free margins, there is no guarantee that the lesion was completely extirpated, as evidenced by 16% of our patients who, even with free margins, had residual disease in hysterectomy.^23^

Unlike our study, in which we found a prevalence of 8% of coexistence of adenocarcinoma in situ and squamous lesions (4 cases), the literature describes that in up to 50% of cases in situ adenocarcinoma can coexist with preinvasive squamous lesions or invasive carcinoma.^32^

Based on a meta-analysis that included 33 studies with 1287 patients, the average age of diagnosis of adenocarcinoma in situ is 36.9 years old, below that found in our study.^33^ However, in Brazilian studies, the mean age of diagnosis of adenocarcinoma in situ is 49 years old, more similar to that found in the present study, which may indicate that early diagnostic methods are not as effective here as in developed countries.^34^

Risk factors for cervical adenocarcinoma are the same for squamous carcinoma, HPV being the most famous of them, especially subtype 18.^35^ Data such as number of partners and condom use are controversial in the literature because HPV is an important confounding factor.^36^

The use of exogenous estrogen therapy is a risk factor known in the literature for both adenoid and cervical carcinoma.^37^ Exposure to estrogens is implicated not only in the metaplasia process, but also in the particular susceptibility of the transformation zone to the evolution of neoplastic lesions.^38^

Unlike what we know for cervical squamous carcinoma, smoking does not appear to be a risk factor for adenocarcinoma, which can be seen in our study, since only 4 (13%) patients were smokers or former smokers.^39^

Early cervical cancer is often asymptomatic, while locally, advanced disease can cause symptoms, the main one being abnormal uterine bleeding, corroborating what was found in the present study.^14^

## Conclusion

The prevalence of adenocarcinoma in situ is increasing, and yet its conduct remains controversial. There is still a large part of the literature that advocates the use of conservative treatment for this disease, even knowing that it is a multifocal disease and may be present even in situations in which the anatomopathological evidence free margins. Given these characteristics, most advocate that hysterectomy remains as the preferred treatment in women who have completed their reproductive purposes. There is benefit of HPV DNA follow-up, especially in those patients who underwent a conservative surgical procedure. Finally, it is observed that there is still a great difficulty in the screening of cervical adenocarcinoma, thus, these constitute a real challenge for the clinician, being urgent the need to implement research studies aimed at facilitating the diagnosis and monitoring of this pathology.
